# Adipocyte Mitochondria: Deciphering Energetic Functions across Fat Depots in Obesity and Type 2 Diabetes

**DOI:** 10.3390/ijms25126681

**Published:** 2024-06-18

**Authors:** Snehasis Das, Alpana Mukhuty, Gregory P. Mullen, Michael C. Rudolph

**Affiliations:** 1 Harold Hamm Diabetes Center, Department of Biochemistry and Physiology, The University of Oklahoma Health Sciences Center, Oklahoma City, OK 73104, USA; 2 Department of Zoology, Rampurhat College, Rampurhat 731224, India

**Keywords:** white adipocyte, brown adipocyte, beige adipocyte, obesity, type 2 diabetes, mitochondrial dysfunction, adipocyte browning, thermogenic adipocyte

## Abstract

Adipose tissue, a central player in energy balance, exhibits significant metabolic flexibility that is often compromised in obesity and type 2 diabetes (T2D). Mitochondrial dysfunction within adipocytes leads to inefficient lipid handling and increased oxidative stress, which together promote systemic metabolic disruptions central to obesity and its complications. This review explores the pivotal role that mitochondria play in altering the metabolic functions of the primary adipocyte types, white, brown, and beige, within the context of obesity and T2D. Specifically, in white adipocytes, these dysfunctions contribute to impaired lipid processing and an increased burden of oxidative stress, worsening metabolic disturbances. Conversely, compromised mitochondrial function undermines their thermogenic capabilities, reducing the capacity for optimal energy expenditure in brown adipocytes. Beige adipocytes uniquely combine the functional properties of white and brown adipocytes, maintaining morphological similarities to white adipocytes while possessing the capability to transform into mitochondria-rich, energy-burning cells under appropriate stimuli. Each type of adipocyte displays unique metabolic characteristics, governed by the mitochondrial dynamics specific to each cell type. These distinct mitochondrial metabolic phenotypes are regulated by specialized networks comprising transcription factors, co-activators, and enzymes, which together ensure the precise control of cellular energy processes. Strong evidence has shown impaired adipocyte mitochondrial metabolism and faulty upstream regulators in a causal relationship with obesity-induced T2D. Targeted interventions aimed at improving mitochondrial function in adipocytes offer a promising therapeutic avenue for enhancing systemic macronutrient oxidation, thereby potentially mitigating obesity. Advances in understanding mitochondrial function within adipocytes underscore a pivotal shift in approach to combating obesity and associated comorbidities. Reigniting the burning of calories in adipose tissues, and other important metabolic organs such as the muscle and liver, is crucial given the extensive role of adipose tissue in energy storage and release.

## 1. Introduction

Adipose tissue (AT) is one of the major metabolic organs in the human body, possessing specialized capabilities for lipid handling. White adipose tissue (WAT) and brown adipose tissue (BAT) are the two primary forms of adipose tissue, with opposing functions. Both white and brown adipocytes are descended from mesenchymal stem cells; however, they originate from distinct lineages that express unique markers. Genetic lineage tracing studies reveal that white adipocytes are descended from the progenitors of Myf5-negative (PDGFRα+, CD29+, and CD44+) mesenchymal stem cells, while brown adipocytes descend from progenitors of Myf5+ (CD34+/CD29, MYF5-, and PAX3+) [1,2,3,4]. BAT can dissipate excess energy to maintain body heat via non-shivering thermogenesis, using mitochondrial uncoupling of electron transport and oxidative phosphorylation. Conversely, WAT adipocytes take up fatty acids and glucose, and synthesize triacylglycerols to store excess energy. When exposed to appropriate stimuli, such as cold exposure or β-adrenergic agonists, WAT remodels into beige adipose tissue (BeAT), which is distinguished by having more mitochondria and a multilocular lipid droplet morphology. Thus, beige adipocytes are found in WAT, and when exposed to cold, they remodel to metabolize substrates to produce heat, a process known as cold-induced thermogenesis [5] (Table 1).

Excess AT deposition and adipocyte expansion in both subcutaneous and visceral regions, including the intra-abdominal cavity, are characteristic of obesity [6,7]. Visceral adipose tissue (VAT) also performs crucial roles in energy regulation, storing energy in the form of triacylglycerols and releasing it in response to increased demand [8,9]. Asymmetry in the availability of substrates, such as imbalanced proportions of carbohydrates, fat, or protein, can cause mitochondrial dysfunction, which affects oxidative respiration and cellular energy production [10]. Despite having a relatively modest density, adipocyte mitochondria play a crucial role in various cellular metabolic processes such as adipogenesis, lipogenesis, and lipolysis, which are deregulated during mitochondrial dysfunction, invariably changing the energetic dynamics of adipocytes [10,11]. It has been shown that metabolic diseases, including T2D and obesity, are strongly linked to adipocyte mitochondrial dysfunction [12,13].

Both shivering and non-shivering thermogenesis produce heat; however, non-shivering thermogenesis is predominantly observed in brown and beige adipocytes [14]. Thermogenesis is energy-demanding and has been the subject of extensive research for its potential in body weight control and improvement to metabolic health. Finding ways to activate beige adipocytes is an appealing therapeutic strategy because activated BeAT in rodents promotes obesity resistance and weight loss [15]; however, further research is needed to determine whether this is feasible in clinical practice. Thermogenesis requires the uptake of glucose, amino acids, and lipids for energy substrates to maintain the increased demand by the mitochondria. For example, in response to cold exposure, brown and beige adipocytes take up circulating free fatty acids, acylcarnitines, and all classes of lipoprotein complexes [15]. Beyond their direct contribution to thermogenesis as a fuel source, lipids play crucial roles in membrane composition, post-translational modifications, and as signaling molecules [16]. Outside the cell, circulating lipid abundance signals the availability of stored energy, a feature that is frequently imbalanced in obesity and T2D [17].

When stimulated by cold, mitochondria in brown and beige adipocytes adopt distinctive morphologies and inter-organelle interactions [18]. The mitochondrion is the site for Uncoupling protein 1 (UCP1) function, as well as fatty acid β-oxidation enzymes, and is therefore at the center of thermogenic control and lipid processing. UCP1 promotes proton diffusion into the mitochondrial matrix without coupling proton movement to ATP (Adenosine triphosphate) synthase activity, which is the chief mechanism for heat production in thermogenic adipocytes. As protons diffuse into the mitochondrial matrix, potential energy in the uncoupled proton gradient is transformed into heat [19]. Because of the depletion of the inner mitochondrial proton gradient and fruitless oxidative cycles, increased cellular energy expenditure occurs, thereby burning nutrient substrates [20]. Accordingly, driving non-shivering thermogenesis in BAT and BeAT may provide an effective means to increase overall energy expenditure and improve whole-body metabolism.

Adipocyte differentiation, lipid metabolism, insulin sensitivity, oxidative capability, and thermogenesis are all negatively impacted by obesity-associated mitochondrial dysfunction, which ultimately leads to metabolic disorders. Table 2 summarizes the major findings in the field over the last two decades and highlights the mechanisms underpinning these findings. Recent research has demonstrated that thiazolidinedione, mitochondria-targeted antioxidants, dietary natural compounds such as berberine, resveratrol, and epigallocatechin-3-gallate, exercise, and caloric restriction improve mitochondrial function [11]. This can help to maintain metabolic homeostasis by causing adaptive thermogenesis of BAT and browning of WAT [11]. Hence, it is essential to understand, in detail, the factors underpinning metabolic dysfunction in WAT, BAT, and BeAT depots that might precede progression to obesity and insulin resistance.

## 2. White Adipocyte Function

WAT contains large adipocytes with single large lipid droplets and fewer mitochondria than BAT or BeAT adipocytes. Although the number of mitochondria is low in WAT, white adipose integrates nutritional and hormonal cues from the blood and its local microenvironment. The WAT metabolic response is either to oxidize incoming fatty acids and carbohydrate fuels through the tricarboxylic acid cycle (TCA) cycle and respiratory chain, or store these fuels safely in the form of triacylglycerols until whole-body energy requirements signal for their release [23]. By controlling lipogenesis and lipolysis, WAT maintains whole-body insulin sensitivity and energy homeostasis [23].

The distribution of WAT can be categorized into two primary depots: subcutaneous WAT (SAT), which is found beneath the skin, and visceral WAT (VAT), which comprises the omental, mesenteric, retroperitoneal, gonadal, and pericardial depots. With the progression of obesity-associated T2D, a decline in the number of mitochondria in mature white adipocytes and adipocyte hypertrophy diminishes the metabolic capability of the mitochondria [46]. The relationship between the two depots and the emergence of insulin resistance (IR) is the focus of much-needed research [47,48].

IR and poor glycemic control are strongly correlated with the percentage of VAT in obese individuals in humans, and surgical ablation of VAT has been shown to slow the onset of T2D in animal models [49,50,51]. Conversely, an increase in SAT mass is protective and negatively correlates with the risk of developing T2D, insulin resistance, and glucose intolerance. People with obesity have lower SAT, which worsens insulin response and encourages the development of T2D [50,52]; therefore, at the same body mass index, insulin-sensitive obesity is associated with a smaller VAT depot, implying a greater proportion of SAT mass. Although they perform different functions, both SAT and VAT contribute to the development of IR. In general, an increase in VAT is associated with a decline in metabolic status, whereas an increase in SAT is associated with improved metabolic status [53]. A difference in mitochondrial content has also been observed between VAT and SAT in both rat and obese individuals, with VAT showing higher mitochondrial content compared to SAT [54,55].

## 3. Mitochondrial Dysfunction in White Adipocytes in Obesity

**Figure 1:**
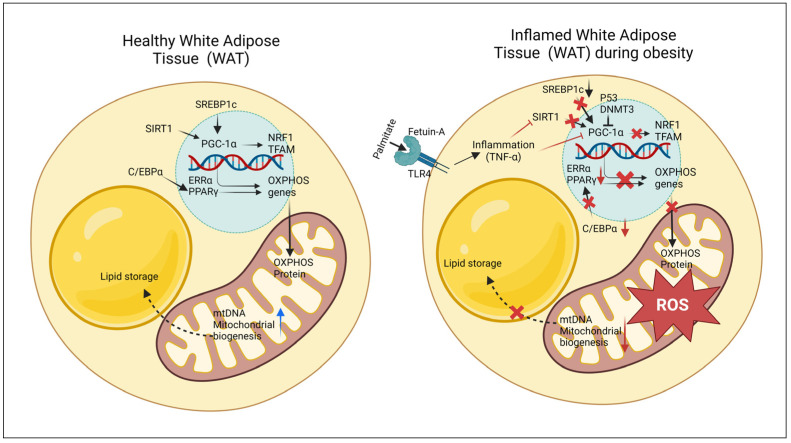
Schematic diagram of mitochondrial regulation in healthy and inflamed white adipocytes. Mitochondrial biogenesis and bioenergetics are regulated by a complex network of transcription factors and activators, including PGC1α and TNF-α, which are influenced by mitochondrial substrate load, ROS, and other factors. Black arrows, downward red arrows, upward blue arrows, T arrows, and red forks represent induction, reduction, increase, inhibitory input and pathway suppression, respectively. Illustration made in BioRender. SIRT1, Silent mating type information regulation 2 homolog 1 (Sirtuin 1); SREBP1c, Sterol regulatory element-binding protein 1c; ERRα, Estrogen-related receptor α; TFAM, Mitochondrial transcription factor A; NRF1, Nuclear respiratory factor 1; OXPHOS, Oxidative phosphorylation; TLR4, Toll-like receptor 4; TNF-α, Tumor necrosis factor α; P53, Tumor protein 53; DNMT3, DNA methyl transferase 3; PPARγ, Peroxisome proliferator-activated receptor γ; C/EBPα, CCAAT/Enhancer-binding protein α; PGC1α, Peroxisome proliferator-activated receptor γ coactivator 1 α.

Several studies have shown that development of T2D and mitochondrial depletion in WAT are associated [56,57]. Obesity-associated mitochondrial dysfunction occurs at different levels. Mitochondrial copy number and content of mitochondrial OXPHOS (Oxidative phosphorylation) enzymes are reduced significantly, which is associated with reduced mitochondrial fatty acid oxidation and ATP production, resulting in adipocyte hypertrophy [58]. Mitochondrial biogenesis and bioenergetics are controlled by a complex network of transcription factors and activators, and PGC1α (Peroxisome proliferator-activated receptor γ coactivator 1 α) is the major regulator of the mitochondrial system. PGC1α activates the nuclear-encoded transcription factor, NRF1 (Nuclear respiratory factor 1), which activates the mitochondrial transcription factor, TFAM (Mitochondrial transcription factor A), to stimulate mitochondrial DNA (mtDNA) replication and transcription for mitochondrial biogenesis as depicted in Figure 1 [59,60]. All these important transcription factors and co-factors are significantly impaired in the inflamed and hypertrophied adipocytes of obese individuals [58,61,62]. Mice with white adipocyte-specific (Adiponectin-Cre) loss of PGC1α develop insulin resistance, associated with loss of thermogenic gene expression in white adipocytes [63]. Microarray profiling studies have shown that, in addition to the key transcriptional regulators PPARγ, ERRα (Estrogen-related receptor α), and PGC1α, numerous mitochondrial genes essential for mitochondrial function and oxidative phosphorylation are downregulated in mouse models, including HFD-induced obese, insulin-resistant, and db/db mice [64].

Increased levels of TNF-α (Tumor necrosis factor α) are the major cause of the obesity-induced decrease in PGC1α expression [65]. Expression of Fetuin-A, a hepato-adipokine, increases under hyperlipidemic conditions, when lipid levels rise in the circulation [66,67]. Elevated levels of palmitate and Fetuin-A are responsible for the stimulation of TLR4 pathway-mediated TNF-α induction observed in hypertrophied, inflamed adipocytes, which may be a crucial mechanism for suppressing PGC1α, as well as mitochondrial biogenesis and fatty acid oxidation [68]. Activated PGC1α and functional mitochondrial levels are also maintained by SIRT1 (Sirtuin 1) and AMPK (AMP-activated protein kinase), which were decreased in obese adipocytes due to lipid-induced inflammation [68,69]. In 3T3-L1 adipocytes, high glucose and free fatty acid concentrations have been shown to directly trigger mitochondrial dysfunction [70]. Additionally, the transcription factors P53 and DNMT3 (DNA methyl transferase) negatively regulate PGC1α-mediated mitochondrial biogenesis and bioenergetics in hypertrophied adipocytes [71].

The production of Reactive Oxygen Species (ROS) and oxidized lipid species, endoplasmic reticulum (ER) stress, and hereditary factors are other major causes of mitochondrial dysfunction, all of which are exacerbated by excessive caloric intake [72]. Cell types that are most susceptible to lipotoxicity experience ectopic lipid accumulation when mitochondrial activity is disrupted, such as steatosis in the liver [72]. ROS generation rises as obesity develops. Since mtDNA does not have nuclear DNA repair mechanisms, it is vulnerable to damage from ROS generated through OXPHOS. As a result, compared to nuclear DNA (nDNA), the mutation rate in mtDNA is much greater, leading to mtDNA polymorphisms resulting from OXPHOS mutation [73].

Recent studies have also highlighted the importance of noncoding RNAs, which act as regulators of the differentiation, development, and function of adipocytes. Deletion of miR155 in an HFD mouse model of diet-induced obesity reduced VAT and exacerbated adipose tissue fibrosis, while suppression of miR-199a/214 in brown and beige adipocytes increased mtDNA copy number. These reports suggest an important connection between micro-RNAs and a potential link between obesity and mitochondrial lipid metabolism [74,75].

During HFD exposures, increased lipid uptake into adipocytes results in an increased mitochondrial substrate load, which then results in increased ETC (Electron transport chain) activity and ROS production [21]. NRF2 (Nuclear respiratory factor 2) and FOXO (Forkhead family of transcription factors) upregulate the expression of antioxidant enzymes as a compensatory mechanism to preserve the intracellular redox state. When the antioxidant defense capacity is overwhelmed by high ROS generation, oxidative stress results in macromolecule oxidation [76]. Mitochondrial-generated ROS also sends an integrated physiological “distress” signal to WAT [76], which is controlled in part by nutrition, hormones, and oxygen fluxes in the tissue [77]. Thus, oxidative stress is one mechanism that accounts for adipocyte dysfunction by impairing insulin signaling and promoting inflammation, which impairs mitochondrial function in WAT [78,79,80]. Obesity is associated with a strong suppression of the expansion of preadipocyte populations in the abdominal adipose depot, which differentiate into white adipocytes to cope with excess energy substrates, such as fats, carbohydrates, or amino acids [81]. Preadipocyte differentiation during WAT expansion is significantly depressed by obesity-induced changes in mitochondrial biogenesis [46,82]. Differentiating preadipocytes increase the relative abundance of mtDNA copy number, accompanied by increased mitochondrial genome replication, the nuclear-encoded TFAM, and elements of deoxynucleotide metabolism necessary for mtDNA replication [83,84,85]. Interestingly, through small interfering RNA (siRNA)-mediated knockdown of TFAM in 3T3-L1 adipocytes, Shi et al. observed further reduction of ETC capacity and O_2_ respiratory rates, accompanied by impaired glucose transport. However, a paradoxical increase in insulin-stimulated Akt phosphorylation ensued as well, the underlying reasons for which were not clear [85]. Recent studies have shown that the use of mitochondrial respiratory inhibitors or knockdown of genes involved in mitochondrial biogenesis severely impair preadipocyte differentiation, resulting in decreased triacylglycerol storage capacity in the mature adipocyte, resulting in lipid spillover [85,86]. This was accompanied by decreases in PGC1α, PPARγ (Peroxisome proliferator-activated receptor γ), C/EBPα (CCAAT/Enhancer-binding protein α), and SREBP-1c (Sterol regulatory element-binding protein 1c) expression levels and upregulation of CREB levels [87]. Additionally, preadipocyte proliferation and differentiation are inhibited by obesity-mediated ROS production, which may prevent the establishment of healthy WAT expansion to accommodate the excess nutrient storage load as obesity progresses [29].

Overall, mounting evidence indicates that dysfunctional mitochondrial components in WAT alter numerous metabolic pathways, eventually perturbing glucose and lipid homeostasis throughout the body, making mitochondrial bioenergetics in WAT a potential therapeutic target to offset metabolic diseases.

## 4. Obesity-Induced Changes in Mitochondrial Function in Brown Adipocytes

**Figure 2:**
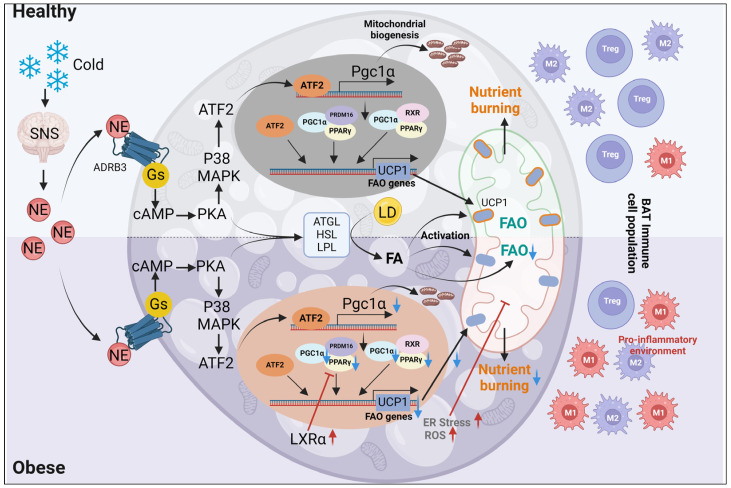
Mitochondrial regulation in brown adipocytes from individuals with or without obesity. Under healthy conditions, mitochondrial thermogenesis in brown adipocytes is triggered by norepinephrine, which stimulates the adrenergic receptor-G_s_ protein-adenylyl cyclase-cAMP-PKA transduction pathway for lipolysis, leading to Ucp1 expression and dissipation of excess energy. In the obese condition, BAT development and function are often defective due to the impairment of preadipocyte differentiation, immune cell infiltration, inflammatory cytokine production, ROS, inappropriate cell death, and ER stress. Black colored arrows, downward blue arrows, upward red arrows, and red T arrows represent activation, reduction, upregulation, and inhibition, respectively. Illustration made in BioRender. SNS, Sympathetic nervous system; NE, Norepinephrine; ADBR3, Adrenergic receptor β-3; Gs, Stimulatory G protein; cAMP, Cyclic adenosine monophosphate; ATF2, Activating transcription factor 2; MAPK, Mitogen-activated protein kinase; PKA, Protein kinase A; RXR, Retinoid X receptor; LXRα, Liver X receptor α; ATGL, Adipose triglyceride lipase; HSL, Hormone-sensitive lipase; LPL, Lipoprotein lipase; LD, Lipid droplet; FA, Fatty acid; FAO, Fatty acid oxidation; BAT, Brown adipose tissue; Treg, Regulatory T cell; ROS, Reactive oxygen species; ER, Endoplasmic reticulum; PPARγ, Peroxisome proliferator-activated receptor γ; PGC1α, Peroxisome proliferator-activated receptor γ coactivator 1 α; PRDM16, PR domain containing 16; UCP1, Uncoupling protein 1.

BAT is chiefly responsible for the energy expenditure needed for thermogenesis to maintain body temperature. BAT thermogenesis occurs in numerous tightly-packed mitochondria that contain the uncoupling protein 1 (UCP1) inner membrane protein, which is highly expressed in brown adipocytes [88]. Norepinephrine secreted from the sympathetic nervous system triggers the canonical adrenergic receptor-G_s_ protein-adenylyl cyclase-cAMP-PKA (Protein kinase A) transduction pathway that stimulates lipolysis. The free fatty acids released then act as both the fuel for β-oxidation and activators of UCP1, which uncouples aerobic respiration by dissipating the inter-membrane proton-motive force (Figure 2); however, it has recently been shown that lipolysis is not necessary for UCP1 activation in BAT [88,89,90]. Nevertheless, an ineffective cycling of ions produces heat rather than ATP [91]. The oxygen consumption by BAT, which accounts for around 2% of body weight, is roughly twice the basal metabolic rate for the entire body under thermoneutral conditions [92]. UCP1 is most abundant in brown adipocyte mitochondria, and brown adipocytes can be distinguished from white adipocytes by higher levels of expression of type 2 iodothyronine deiodinase (DIO2) and the transcription co-regulators PRD-BF-1-RIZ1 homologous domain containing protein 16 (PRDM16), PGC1α, and regulator of lipolysis Cidea [93]. PRDM16, a transcriptional co-activator of PGC1α, combines to upregulate genes involved in mitochondrial biogenesis, uncoupling, and oxidative phosphorylation, a key element for mitochondrial biogenesis in BAT [31]. Thus, the PRDM16–PGC1α–NRF1/2–TFAM axis is critical for the regulation of mitochondrial number and function in BAT.

Adipocytes deficient in TFAM have reduced levels of mitochondrial oxidative phosphorylation complex proteins and increased inflammation, resulting in the ‘whitening’ of brown adipocytes [94,95]. PGC1α is the key downstream target of adrenergic receptor activation-mediated BAT thermogenesis [96], which in turn activates numerous nuclear and non-nuclear factors, including retinoid receptors (RXRs), thyroid hormone receptors (THRs), other PPARs (PPARα/β/δ), glucocorticoid receptors, estrogen-related receptors (ERRs), forkhead box O1 (FOXO1), and the liver X receptor (LXR). By doing so, PGC1α activity strongly modulates mitochondrial energy metabolism [97]. An obesity-associated increase in LXRα is inversely associated with decreased Ucp1 expression in BAT [98]. Mechanistically, LXRα helps to recruit RIP140 to displace PPARγ from the enhancer region of the Ucp1 promoter because the RIP140 binding site overlaps with the PPAR response element in this promoter region [97]. The vitamin D receptor (VDR) is another nuclear receptor important for maintaining adipocyte thermogenesis by directly inhibiting adipocyte Ucp1 expression. This is evidenced by the observation that VDRKO mice are resistant to diet-induced obesity due to increased expression of Ucp1, 2, and 3, as well as stimulated fatty acid oxidation in both WAT and BAT [99,100], and overexpression of VDR in adipocytes reduces thermogenesis in WAT and BAT, leading to obesity [101].

Another important mechanism controlling the BAT thermogenic program is the recruitment of immune cells and pro-inflammatory cytokine cell-to-cell communication [102]. A good example is alternately activated (M2) macrophages. Macrophages are essential for maintaining a cold-adaptive thermogenic program in BAT, most likely because of their capacity for catecholamine production [103]. Besides their role in maintaining thermogenesis in BAT, M2 macrophages induce white adipocyte browning by clearing dead adipocytes and stimulating recruitment of PDGFRα-positive adipocyte precursor cells upon activation of the β3-adrenergic receptor [104]. Obesity-associated suppression of M2 macrophages affects both BAT thermogenesis and browning of WAT [105]. Beyond macrophages, genetic ablation of T cells reduces thermogenic marker expression and enhances the invasion of macrophages that promote inflammation [106]. In obesity, BAT exhibits a higher level of chronic inflammation, which is typified by the release of cytokines, T cell infiltration, a reduction of regulatory T cells, and pro-inflammatory M1 macrophages. However, in contrast to white adipose depots, BAT takes longer to manifest the inflammatory condition [107,108]. Elevated invasion of M1 macrophages and pro-inflammatory cytokines produced in BAT cause a decrease in UCP1 expression, which decreases thermogenic activity [109]. Furthermore, pro-inflammatory immune cells that have infiltrated the body, as well as circulating and locally synthesized chemokines, strongly decrease BAT recruitment in individuals with obesity [110].

Pattern recognition receptors such as NOD2 (Nucleotide-binding oligomerization domain-containing protein 2) and TLR2 (Toll-like receptor 2), which are upstream of the NF-κB (Nuclear factor kappa-light-chain-enhancer of activated B cells) pro-inflammatory pathway, exhibit a time-dependent downregulation of expression during normal brown preadipocyte development [111]. In obesity, brown preadipocyte development and brown adipogenesis are suppressed in an NF-κB-dependent manner, when these receptors are normally activated by their respective agonists [34]. Similarly, brown differentiation is inhibited in vitro by pro-inflammatory molecules released by T cells and macrophages, such as TNF-α, IL-1 (Interleukin 1), LPS (Lipopolysaccharides), and Oncostatin M [37,38,39]. In these studies, pro-inflammatory factors downregulated key adipogenic regulators including PPARγ, which replicated effects observed earlier in studies of white preadipocyte differentiation [112]. Additionally, inflammatory signals may encourage cell death that can hinder BAT growth. For example, TNF-α-induced apoptosis has been reported in both brown and white adipocytes [39,113,114].

An increased requirement for protein folding in obesity can lead to ER stress in BAT, which in turn activates the unfolded protein response (UPR). Briefly stated, the UPR attempts to repair the ER through three pathways: reducing protein translation, promoting protein folding, and, if the repair process is unsuccessful, inducing apoptosis. Bip (Binding immunoglobulin protein), Chop (C/EBP homologous protein), Atf4 (Activating transcription factor 4), and Atf6 (Activating transcription factor 6) are key indicators of UPR, which are overexpressed in BAT from obese mice fed an HFD [107,115]. Conversely, oxidative stress indicators were also observed in the BAT of obese mice, in that elevated ROS generation and a decrease in antioxidant capacity in BAT were observed [107]. The dual effects of ROS as pro-oxidative molecules at high pathological concentrations, when they exceed the antioxidant defense, and ROS action as secondary messengers in cell signaling processes at physiological levels, explain the relevant role of oxidative stress in BAT function [35,36].

Together, BAT development and function are often defective in obesity/T2D due to impairment of preadipocyte differentiation into brown adipocytes, immune cell infiltration, inflammatory cytokine production, inappropriate cell death, and ER stress, as depicted in Figure 2 [103,105,106,107]. BAT thermogenesis plays a crucial role in energy expenditure and metabolic regulation, but its function is often compromised in obesity and T2D. In obesity, increased adiposity and inflammatory cytokines disrupt BAT thermogenesis by reducing the expression of UCP1, essential for heat production [88,93]. This downregulation of UCP1 diminishes the effectiveness of mitochondrial uncoupling, leading to decreased energy dissipation and metabolic dysfunction. Moreover, in T2D, dysregulated glucose and lipid metabolisms contribute to mitochondrial dysfunction in BAT, characterized by impaired oxidative phosphorylation and increased oxidative stress [107]. Consequently, dysfunctional mitochondria in BAT exacerbate metabolic disturbances, impairing thermogenic capacity (energy expenditure) and worsening insulin resistance. Understanding the interplay between BAT thermogenesis and mitochondrial dysfunction in obesity and type 2 diabetes is critical for developing targeted therapies to improve metabolic health and combatting these widespread metabolic disorders.

## 5. Mitochondrial Impairments in Beige Adipocytes during Obesity

**Figure 3:**
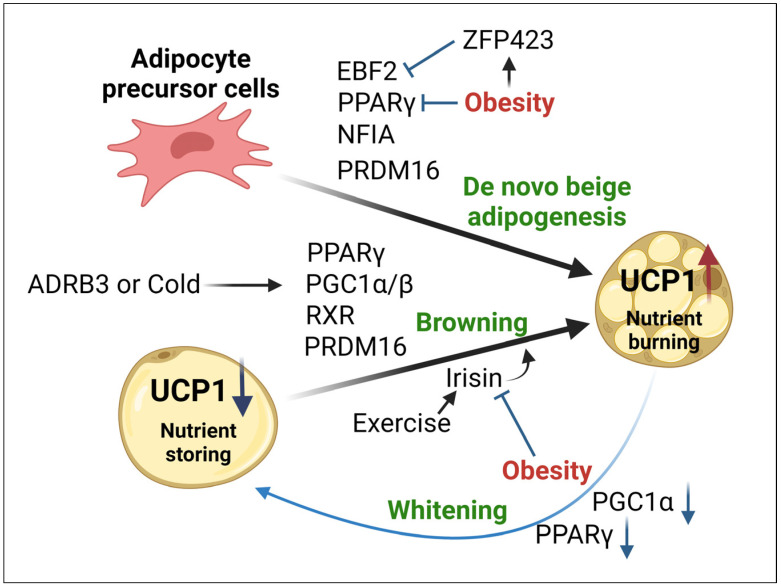
Diagram showing the regulation of de novo beige adipogenesis and white adipocyte browning. β-adrenergic receptors (β-ARs) are activated by cold exposure, leading to browning of WAT to increase thermogenesis. Obesity is associated with a significant decrease in PGC1α expression, reducing the browning of white adipocytes, and inhibition of major regulators such as EBF2 and PPARγ, reducing de novo beige adipogenesis from precursor cells. Black colored arrow, upward red arrows, downward blue arrows, and T arrows represent activation, upregulation, reduction, and inhibition, respectively. Illustration made in BioRender. ADRB3, Adrenergic receptor β-3; RXR, Retinoid X receptor; PPARγ, Peroxisome proliferator-activated receptor γ; PGC1α/β, Peroxisome proliferator-activated receptor γ coactivator 1 α/β; PRDM16, PR domain containing 16; UCP1, Uncoupling protein 1; EBF2, Early B cell factor 2; ZFP423, Zinc figure protein 423; NFIA, Nuclear factor I A.

Emerging evidence indicates that WAT contains a unique type of adipocyte known as beige adipocytes. These adipocytes are characterized by greater mitochondrial number and a retained capacity for nutrient burning. When β-adrenergic receptors (β-ARs) are activated during cold exposure, “browning of WAT” occurs, producing more mitochondria, a multilocular morphology, and UCP1-positive beige adipocytes from white adipocytes to increase thermogenesis [116,117]. There is ongoing debate as to the origin of beige adipocytes [118,119]. Three independent pathways leading to the formation of beige adipocytes have been identified: (a) mature white adipocytes remodel into beige adipocytes; (b) an ASC-specific subtype precursor exists, forming beige adipocyte populations; and (c) beige adipocytes originate from de novo differentiation of non-specific tissue-resident ASC progenitors. Interestingly, PRDM16 protein stabilization by TZDs causes white-to-brown adipose tissue conversion, indicating PRDM16 as a crucial “browning” determining factor [120]. Finally, recent studies have identified a new exercise-induced PGC1α-dependent myokine, irisin, that stimulates WAT to develop in a manner similar to BAT and enhances effective skeletal muscle–WAT interactions [30,32]. In a UCP1-mediated mechanism, irisin can directly stimulate the “beiging” of white adipocytes, as shown in Figure 3.

Irisin levels are significantly decreased in obese, diabetic, and sedentary animal models, affecting beige fat development and adaptive thermogenesis [121]. It is widely recognized that obesity causes both BeAT and BAT to undergo “whitening”, in which beige and brown adipocytes acquire the unilocular lipid droplet morphology characteristic of white adipocytes, losing their beige and brown thermogenic metabolic properties [122,123]. The transcriptional coactivators PGC1α and PGC1β play a crucial role in the molecular network that regulates mitochondrial biogenesis in both WAT and BAT [124,125,126]. Compared to wild-type mice, adipocyte-specific (Adiponectin-Cre) Pgc1α-deleted mice showed decreased uncoupling of O_2_ respiration and were more prone to glucose intolerance and insulin resistance. PGC1α can direct the adipogenic master regulator, PPARγ, from a white adipocyte program to a beige program linked to the dissipation of energy by increasing UCP1 expression [127]. In individuals with morbid obesity, a significant decrease in PGC1α expression in SAT is thought to reduce white adipocyte browning, although it is unclear whether the decrease in PGC1α was the cause or consequence of obesity [128]. Some reports have suggested that adrenergic stimulation of BAT thermogenesis or regulation of BAT function may be promising treatments for T2D and obesity [95,129,130]. Gaining further insights into the molecular processes that regulate the browning of adipocytes could pave the way for the development of pharmacological interventions to prevent or reduce obesity and offset its associated metabolic issues. As such, recent studies have reported strategies to facilitate the development of pharmaceutical approaches to raise energy expenditure as a means of addressing obesity and metabolic disorders [131,132,133].

In WAT, there is invariably a beige-to-white shift and mitochondrial breakdown following the removal of the cold or β3-AR agonist stimuli [134], and the characteristic increases in oxidative phosphorylation complexes and levels of UCP1 are rapidly reversed and a white adipocyte morphology re-emerges [135]. Gene annotation enrichment analysis during the transition from beige to white adipocytes revealed that the most significantly enriched genes were associated with mitochondrial cellular components, the ETC, and biological processes related to oxidation and reduction [5].

It is important to note that, in beige adipocytes, a UCP1-independent thermogenesis pathway has been recently identified. Thermogenesis that is independent of UCP1 is regulated by Ca^2+^ cycling mediated by SERCA2b (Sarco/Endoplasmic reticulum Ca^2+^-ATPase 2b) [135]. In this setting, enhanced Ca^2+^ cycling induced by α1- and/or β3-adrenergic receptor activation or the SERCA2b-RyR2 pathway enhances UCP1-independent thermogenesis. Beige adipocytes from Ucp1-deficient mice had increased tricarboxylic acid metabolism, glycolysis, and pyruvate dehydrogenase activity to drive ATP-dependent thermogenesis. Moreover, beige adipocytes can be strongly induced in UCP1-knockout mice by the β3-AR agonist CL316,243, and direct mitochondrial breakdown occurs after withdrawal of the browning stimulus [134].

Impaired Ca^2+^ homeostasis, decreased ATP synthesis, increased ROS generation, impaired mitochondrial enzyme activity, and anomalies in systemic energy in obese adipocytes are all consequences of dysregulated mitochondrial dynamics, which have a negative impact on mitochondrial function [41,42]. According to recent reports, loss of mitochondrial dynamics due to mitochondrial fusion regulators Mfn2 (Mitofusin-2) and Opa1 (Optic atrophy 1) in 3T3-L1 preadipocytes induces accumulation of intracellular triacylglycerols [43,44,136]. In vivo, severe structural abnormalities of the mitochondria and poor adaptive thermogenesis were observed in adipocyte-specific Mfn2-deficient mice [137,138]. Reduced MFN2 expression has been linked to decreased mitochondrial function in subcutaneous and visceral adipose tissues of individuals with obesity [139]. The study also found that obesity and insulin resistance can be induced by adipocyte-selective inducible ablation of Mfn2 in mice [139]. It is unclear how mitochondrial dynamics affect adipocyte metabolism, and further research is needed to elucidate the individual regulatory mechanisms of mitochondria in white, brown, and beige adipocytes that may be targeted to combat obesity and T2D.

Although BeAT is highly abundant in the postnatal period, when maternally supplied nutrients are oxidized, in part, to preserve body heat [140,141], recent research indicates that WAT contains both beige and white Adipocyte stem-like Cells (ASCs), which are established in neonates and determined in committed ASC subtypes [5,118,142]. In mice and humans, selectively increasing and/or prolonging BeAT increased energy expenditure and improved insulin sensitivity and glucose control when challenged with an obesogenic diet [14,33,120,131,132,133,143,144]. Thus, mature beige adipocytes initially arise de novo from ASCs and remain primed to burn nutrients and protect against diet-induced obesity [118]. Our lab has recently shown that perinatal exposure to high levels of omega-6 fatty acids reduces fatty acid oxidation in the ASCs of postnatal 12-day-old pups, which was associated with a white adipocyte morphology in inguinal SAT at a point in life when beige adipocytes should be highly prevalent [145]. Interestingly, the Nuclear Receptor 2 Family member 2 (NR2F2, also known as COUPTFII), which is a key metabolic regulator, was reduced in the ASCs from pups exposed to high levels of omega-6 fatty acids. Furthermore, ligand activation of NR2F2 in undifferentiated ASCs induced gene expression of the critical beige regulators Prdm16, Pparg, and C/ebpa, suggesting a role for NR2F2 in determining beige adipocyte commitment early in life. It is possible that by identifying the regulators that control fate determination in beige adipocytes, novel targets will be identified that can improve mitochondrial metabolic dysfunction in individuals with obesity and T2D.

## 6. Conclusions

The growing body of literature in mitochondrial research, particularly addressing adipocyte metabolism, underscores the significant role mitochondria play in the development and progression of obesity and associated metabolic comorbidities. Adipose tissue exhibits remarkable plasticity, capable of undergoing significant expansion, reduction, or transformation in response to specific stimuli such as cold-induced thermogenesis or caloric excess. Research, primarily driven by the need to better understand the function of adipocytes in the context of obesity and related diseases, has yielded promising findings that not only open new avenues for combatting obesity but have also revealed the existence of multipotent stem cells within WAT [146]. It is now widely acknowledged that adipose tissue functions not merely as a passive reservoir to safely harbor excess nutrients but also as a regulator and integrator of the delicate equilibrium between energy intake and energy expenditure, maintaining energy homeostasis. Adipose tissues secrete numerous signals that influence the function of metabolic organs throughout the body, including other fat depots, muscles, and the liver [147,148]. Cumulative findings offer a wide array of potential therapeutic targets, such as modulation of adipocyte differentiation and transformation, regulation of ROS production, and mitochondrial dynamics, which can direct substrates towards energy dissipation. Mitochondria can be targeted therapeutically by different means such as enhancing mitochondrial biogenesis, increasing mitochondrial function, uncoupling, and modulating mitochondrial dynamics. For example, compounds such as metformin and resveratrol stimulate mitochondrial biogenesis and improve overall metabolic health [149,150]. Coenzyme Q10, lipoic acid, omega-3 fatty acids, and TZDs can improve mitochondrial function and increase insulin sensitivity [151,152,153,154]. Mitofusins and dynamin-related protein 1 can regulate mitochondrial dynamics and improve metabolic outcomes [155,156]. Exercise stimulates mitochondrial biogenesis, function, and uncoupling and enhances metabolism [157,158]. Mirabegron, a drug approved for the treatment of overactive bladders, is a β3-adrenergic agonist that shows promise for the treatment of metabolic diseases [159,160]. Although there is ongoing debate about the viability of activating thermogenic adipocytes to improve metabolic status in clinically obese individuals, it is clearly also a great opportunity [161,162,163]. While both BAT and beige fat activation have potential for treating T2D and obesity, beige fat may offer a better solution due to its plasticity, broader distribution, and potential for larger-scale remodeling within WAT. These attributes make beige fat a versatile and potentially more effective target for enhancing energy expenditure and improving metabolic health. Further research is necessary to fully understand the mechanisms and develop therapeutic strategies for beige fat activation.

## Figures and Tables

**Table 1:** Table showing the properties of white, brown, and beige adipocytes, including cellular and lipid droplet morphology, function, mitochondrial density, precursor, origin, UCP1 expression, and marker genes.

	White Adipocyte	Brown Adipocyte	Beige Adipocyte
**Function**	Energy storage, Endocrine signaling	Thermogenesis, Endocrine signaling	Adaptive thermogenesis, Endocrine signaling
**Mitochondrial density**	Low	High	Medium
**Origin**	Myf5- progenitors	Myf5+ progenitors	Myf5- progenitors
**Regulators of fate determination**	BMP-4, BMP-10, FGF10, PPARγ, C/EBPs	BMP-7, PRDM16, PPARγ, C/EBPs, PGC1α	BMP-7, PRDM16, PPARγ, C/EBPs, PGC1α
**UCP1 expression**	Low	High	High
**Lipid droplet**	Large unilocular	Small multilocular	Medium multilocular
**Markers**	Leptin, Hoxc8, Hoxc9	UCP1, Zic1, Lhx8	UCP1, Cd137, Tmem26
**Morphology**	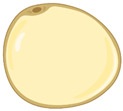	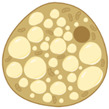	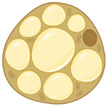

Myf5, Myogenic factor 5; BMP-4/7/10, Bone morphogenetic protein 4/7/10; FGF10, Fibroblast growth factor 10; PPARγ, Peroxisome proliferator-activated receptor γ; C/EBP, CCAAT/Enhancer-binding protein; PRDM16, PR domain containing 16; PGC1α, Peroxisome proliferator-activated receptor γ coactivator 1 α; Hoxc8/9, Homeobox C8/9; UCP1, Uncoupling protein 1, Zic1, Zinc finger protein of the cerebellum 1; Lhx8, LIM Homeobox 8; Cd137, Cluster of differentiation 137; Tmem26, Transmembrane protein 26.

**Table 2:** Summary of mitochondrial deregulation associated with white, brown, and beige adipocyte dysfunction and relevant experimental models.

Functional Implications	Model or Clinical Condition	Citation
**White adipocytes**		
Mitochondria-generated ROS send physiological “distress” signals in WAT during caloric excess	GSTA4-silenced 3T3-L1 adipocytes and GSTA4-null mice	[21]
Recruitment of inflammatory cells into WAT	B6.V-Lep^ob^/OlaHsd mice, CD11c-DTR mice	[22]
WAT stored fuels as triacylglycerols, until whole-body energy demand signals for lipid release	*ob/ob* mice	[23]
Asymmetry in substrate availability causes mitochondrial malfunction	*db/db* mice, *ob/ob* mice	[10]
WAT is highly vascularized and innervated for its endocrine signaling functions	Type 2 Diabetic patients	[24]
WAT can remodel vasculature and extracellular matrix (ECM) to allow tissue expansion, oxygenation, and mobilization of nutrients	*ob/ob* mice, mice fed a high-calorie diet; Type 2 Diabetic patients	[25,26]
WAT expansion occurs through increased adipocyte size (hypertrophy) and/or number (hyperplasia)	Cross of adiponectinPrtTA (adnP-rtTA) transgenic mice with TRE-cre and Rosa26-loxP-stoploxP-lacZ transgenic mice	[27]
Browning of adipocytes blocks lipid spillover	Engrailed-1 (En1)-CreERT-inducible mice crossed with Rosa-floxed Stop-LacZ mouse, Sox10-Cre/Rosa26-YFP model, A-Zip mice	[28]
Adaptive thermogenesis of BAT and browning of WAT maintain metabolic homeostasis in obese diabetic mice	3T3L1 cells, *db/db* mice, *ob/ob* mice, Glut4-knockout mice, Pgc1α-knockout mice, Mfn2-knockout mice	[11]
**Brown adipocytes**		
ROS prevent establishment of healthy BAT by inhibiting preadipocyte proliferation and differentiation	High-Fat Diet (HFD)-fed mice, Obese patients	[29]
PRDM16 protein stabilization by Thiazolidinediones (TZDs) promotes white-to-brown adipose tissue conversion	3T3-F442A cells, CtBP-1+/− CtBP-2+/− and CtBP-1−/− CtBP-2−/− MEFs, 3T3-L1 cells	[30]
Loss of PRDM16 in brown adipose precursors results in the loss of brown adipocyte features	Primary brown pre-adipocytes and myoblasts from P0–P4 Swiss-Webster mice, Cross of Myf5Cre/1 mice18 with rosa26R3 (R26R3)-YFP mice19, Prdm16-knockout mice	[31]
PGC1α stimulates FNDC5 to promote irisin release, which directly stimulates “browning” of white adipocytes	C57BL/6J mice, Obese BALB/c mice	[5,32]
PRDM16 controls thermogenic programming and preserves the fate of brown adipocytes	Myf5Cre, Rosa26Cre mice, Prdm16flox and Prdm3flox mice	[33]
Brown preadipocyte development and adipogenesis are suppressed in an NF-κB-dependent manner	C3H10T1/2 cells	[34]
Diminished or excessive ROS affects BAT recruitment	3T3-L1 cells, adipocytes isolated from lean and obese patients	[35,36]
Brown adipocyte differentiation is inhibited by pro-inflammatory cytokines such as TNF-α, IL-1, LPS, and Oncostatin M, released by T cells and macrophages	Stromal vascular cells from C57BL/6J mice, High-fat diet (HFD)-fed C57BL/6J mice, BAT preadipocytes from C57BL/6J mice, Primary human stromal vascular cells	[37,38,39]
Persistent activation of RalA suppresses energy expenditure in obese adipose tissue	*Rala*^f/f^ (*n* = 8) and *Rala*^AKO^ fed with HFD	[40]
**Beige adipocytes**		
Impaired Ca^2+^ homeostasis, decreased ATP synthesis, increased ROS generation, changed mitochondrial enzyme activity, and anomalies in systemic energy negatively impact mitochondrial function in BeAT	Adipocytes from C57BL/6J mice, 3T3-L1 cells, Adipose tissue from obese patients	[41,42]
When stimulated by cold, mitochondria in brown and beige adipocytes adopt distinctive morphologies and inter-organelle interactions	C57BL/6J mice	[18]
Non-shivering thermogenesis is mostly found in brown and beige adipocytes	β(3)-adrenoceptor-knockout mice, human obese patients, 3T3L1 cells	[14]
Loss of mitochondrial dynamics due to Mfn2 or Opa1 knockdown induces buildup of intracellular triacylglycerols in adipocytes	Adipose tissue from obese patients, HFD mice	[43,44]
Impairment of phosphorylation of DRP1 at serine 600 by protein kinase A (PKA) impairs adipocyte function	Adipocytes from C57BL/6J mice, GF gnotobiotic mice	[45]

GSTA4, Glutathione S-transferase alpha 4; ROS, Reactive oxygen species; WAT, White adipose tissue; ob/ob, leptin-deficient obese mice; db/db, leptin-receptor-deficient mice; ECM, Extracellular matrix; En1, Engrailed-1; GLUT4, Glucose transporter type 4; Mfn2, Mitofusin-2; BAT, Brown adipose tissue; PGC1α, Peroxisome proliferator-activated receptor γ coactivator 1 α; TZDs, Thiazolidinediones; HFD, High-fat diet; PRDM16, PR domain containing 16; FNDC5, Fibronectin type III domain-containing protein 5; PKA, Protein kinase A; BeAT, Beige adipose tissue; Opa1, Optic atrophy 1; DRP1, Dynamin-1-like protein; ATP, Adenosine triphosphate, RalA, RAS like proto-oncogene; TNF-α, Tumor necrosis factor α; IL-Interleukin; LPS, Lipopolysaccharide; NF-κB, Nuclear factor kappa-light-chain-enhancer of activated B cells.

## Data Availability

Not applicable.
